# Exendin-4 blockade of T1R2/T1R3 activation improves Pseudomonas aeruginosa-related pneumonia in an animal model of chemically induced diabetes

**DOI:** 10.1007/s00011-024-01891-8

**Published:** 2024-05-15

**Authors:** Shanjun Yu, Chaoqun Xu, Xiang Tang, Lijun Wang, Lihua Hu, Liang Li, Xiangdong Zhou, Qi Li

**Affiliations:** 1 Department of Respiratory Medicine, The First Affiliated Hospital of Hainan Medical University, Haikou, Hainan 570102 China; 2 Hainan Province Clinical Medical Center of Respiratory Disease, Haikou, Hainan 570102 China; 3 Emergency and Trauma College, Hainan Medical University, Haikou, Hainan 579199 China

**Keywords:** Sweet taste receptor, Diabetes, *Pseudomonas aeruginosa*, Pneumonia, Exendin-4

## Abstract

**Objective:**

Poorly controlled diabetes frequently exacerbates lung infection, thereby complicating treatment strategies. Recent studies have shown that exendin-4 exhibits not only hypoglycemic but also anti-inflammatory properties. This study aimed to explore the role of exendin-4 in lung infection with diabetes, as well as its association with NOD1/NF-κB and the T1R2/T1R3 sweet taste receptor.

**Methods:**

16HBE human bronchial epithelial cells cultured with 20 mM glucose were stimulated with lipopolysaccharide (LPS) isolated from *Pseudomonas aeruginosa* (PA). Furthermore, Sprague‒Dawley rats were fed a high-fat diet, followed by intraperitoneal injection of streptozotocin and intratracheal instillation of PA. The levels of TNF-α, IL-1β and IL-6 were evaluated using ELISAs and RT‒qPCR. The expression of T1R2, T1R3, NOD1 and NF-κB p65 was assayed using western blotting and immunofluorescence staining. Pathological changes in the lungs of the rats were observed using hematoxylin and eosin (H&E) staining.

**Results:**

At the same dose of LPS, the 20 mM glucose group produced more proinflammatory cytokines (TNF-α, IL-1β and IL-6) and had higher levels of T1R2, T1R3, NOD1 and NF-κB p65 than the normal control group (with 5.6 mM glucose). However, preintervention with exendin-4 significantly reduced the levels of the aforementioned proinflammatory cytokines and signaling molecules. Similarly, diabetic rats infected with PA exhibited increased levels of proinflammatory cytokines in their lungs and increased expression of T1R2, T1R3, NOD1 and NF-κB p65, and these effects were reversed by exendin-4.

**Conclusions:**

Diabetic hyperglycemia can exacerbate inflammation during lung infection, promote the increase in NOD1/NF-κB, and promote T1R2/T1R3. Exendin-4 can ameliorate PA-related pneumonia with diabetes and overexpression of NOD1/NF-κB. Additionally, exendin-4 suppresses T1R2/T1R3, potentially through its hypoglycemic effect or through a direct mechanism. The correlation between heightened expression of T1R2/T1R3 and an intensified inflammatory response in lung infection with diabetes requires further investigation.

## Introduction

Diabetes mellitus (DM) is a rapidly growing global endemic disease and is generally accepted as an independent risk factor for lower respiratory tract infections [1, 2]. Accumulating investigations have revealed that compared to a healthy lung, a diabetic lung is more susceptible to pathogenic microorganisms such as gram-negative bacteria, *Mycobacterium tuberculosis* (MT), and even severe acute respiratory syndrome coronavirus 2 (SARS-CoV-2), and individuals with diabetes are also more prone to experiencing severe episodes of pneumonia [3–5]. Specifically, type 2 diabetes mellitus (T2DM) significantly increases the risk of mortality and complications among hospitalized patients with pneumonia, particularly older adults [6, 7]. Although the glycemic index is known to be linked to increased infections in patients with diabetes [8–10], the underlying molecular mechanisms remain unclear.

Taste 1 receptors (T1Rs) belong to the G protein-coupled receptor family and consist of three subunits: T1R1, T1R2, and T1R3 [11]. T1R1 and T1R3 are primarily responsible for sensing umami taste, whereas sweetness is perceived by an obligate heterodimer formed by T1R2 and T1R3 [12, 13]. Therefore, T1R2 and T1R3 are also collectively referred to as the sweet taste receptors (STRs). Previous studies have demonstrated that metabolizable mono- and disaccharides, such as glucose and sucrose, as well as artificial sweeteners such as sucralose and aspartame, can stimulate STRs [13–15]. Intriguingly, although T1R2 and T1R3 were initially identified in taste receptor cells clustered within tongue taste buds, recent studies have shown that these molecules are also highly expressed in the respiratory tract and play important regulatory roles in the airway immune response and pulmonary vascular endothelial permeability [16, 17]. Recently, the results of several studies have indicated that in the presence of sufficient glucose, STRs may inhibit nuclear and mitochondrial Ca^2+^ responses, reducing airway epithelial ciliary oscillation and the production of nitric oxide, thus inhibiting the elimination of pathogens [18, 19]. This phenomenon may be one of the reasons for airway susceptibility to pathogens during hyperglycemia. However, current investigations on STRs predominantly focus on the upper airway, with few studies examining the lower airway. In addition, the role of STRs in the airway inflammatory response and the signaling cascades involved remain unknown.

The occurrence of lung inflammatory storms has been identified as an important contributing factor to the elevated mortality rates observed in diabetic individuals with pneumonia [20]. Under normal circumstances, when various pathogens, including *Pseudomonas aeruginosa* (PA), invade the respiratory tract of the host, some of the molecular patterns they express, such as lipopolysaccharide (LPS), can activate a group of pattern recognition receptors (PRRs) in airway epithelial cells [21]. Activated PRRs further stimulate downstream signaling pathways, such as nuclear factor (NF)-κB, to produce proinflammatory cytokines, including interleukin (IL)-6, IL-1β, and tumor necrosis factor (TNF)-α [22]. The production of these cytokines leads to the accumulation of inflammatory cells, such as lymphocytes, neutrophils, and macrophages, to defend against the invading pathogens [23]. However, previous findings have shown that diabetic patients with pneumonia display excessive production of proinflammatory cytokines, known as ‘cytokine storms’ [24]. The delicate balance between inflammation and anti-inflammation is disturbed, resulting in severe lung damage. Notably, nucleotide-binding oligomerization domain-containing protein 1 (NOD1), a host intracellular PRR, can be activated directly or indirectly by some molecular patterns, including the bacterial peptidoglycan component diaminopimelic acid and LPS, to trigger downstream NF-κB signaling, which substantially contributes to airway inflammation induced by pathogens [25]. Moreover, the available evidence has shown that hyperactivation of NF-κB plays an important role in cytokine storms through multiple mechanisms [20]. However, whether NOD1/NF-κB is overexpressed in lung infection with diabetes remains to be further explored.

The effects of drug treatment for patients with pneumonia and diabetes are disappointing. Nevertheless, certain medications that lower glucose may ameliorate lung inflammation and lung function [26]. Exendin-4 (Ex-4), a glucagon-like peptide-1 receptor (GLP-1R) agonist with hypoglycemic effects, was recently shown to inhibit small intestinal glucose sensing and absorption via repression of T1R2/T1R3 in diabetic mice [27]. Moreover, a recent study showed that the deletion of T1R3 alleviated intestinal inflammation in mice with acute colitis [28]. Therefore, the repression of T1R2/T1R3 caused by Ex-4 may alleviate inflammation in diabetic patients with lung infection. Given these findings, we hypothesized that when the diabetic lung is invaded by a pathogen such as PA, persistent hyperglycemic conditions may induce the upregulation of T1R2/T1R3 and lead to the overexpression of NOD1 via the molecular pattern of a pathogen, resulting in hyperactivation of NF-κB and subsequently the occurrence of cytokine storms. Ex-4, a hypoglycemic drug, may alleviate lung inflammation by inhibiting NOD1/NF-κB and T1R2/T1R3.

This study investigated whether high glucose could increase the overexpression of proinflammatory cytokines (IL-6, IL-1β, TNF-α), T1R2, T1R3, NOD1 and NF-κB p65 in PA-derived LPS-stimulated airway epithelial cells and in the lungs of PA-infected rats with diabetes. Concurrently, we aimed to ascertain whether Ex-4 can mitigate the upregulation of T1R2/T1R3, the overexpression of NOD1 and NF-κB p65 and the overproduction of proinflammatory cytokines in the aforementioned models. The findings of this study may provide promising prospects for future research on alleviating the inflammatory storms associated with pneumonia in patients with diabetes.

## Materials and methods

### Materials

The immortalized human bronchial epithelial cell line 16HBE (Cat. BFN608008569) was purchased from Qingqi Biotechnology (Shanghai, China). LPS from *Pseudomonas aeruginosa* 10 (Cat. L8643) was purchased from Sigma‒Aldrich (Darmstadt, Germany). Exendin-4 (Cat. 141758-74-9) was purchased from MedChemExpress (New Jersey, USA). A Cell Counting Kit (CCK)‑8 (Cat. BS350B) was obtained from Biosharp (Anhui, China). Human TNF-α (Cat. ml077385), IL-1β (Cat. ml058059), and IL-6 (Cat. ml027379) and rat TNF-α (Cat. ml002859), IL-1β (Cat. ml058059), and IL-6 (Cat. ml102828) enzyme‑linked immunosorbent assay (ELISA) kits were obtained from Mlbio (Shanghai, China). An NF-κB activation-nuclear translocation assay kit (Cat. SN368) was purchased from Beyotime Biotechnology (Shanghai, China). The MiniBEST Universal RNA extraction kit (Cat. 9767), PrimeScript RT Reagent Kit with gDNA Eraser (Cat. RR047A), and TB Green Premix Ex Taq II (Cat. RR820A) were obtained from TaKaRa Bio (Beijing, China). Rabbit anti-T1R2 polyclonal (Cat. bs-9599R) and rabbit anti-T1R3 polyclonal antibodies (Cat. bs-23618R) for immunofluorescence and rabbit anti-β-actin polyclonal antibodies (Cat. bs-0061R) were purchased from Bioss (Beijing, China). Rabbit anti-T1R2 polyclonal (Cat. orb336464) and rabbit anti-T1R3 polyclonal (Cat. orb541918) antibodies for western blotting were obtained from Biorbyt (Cambridge, UK). Rabbit anti-NOD1 polyclonal antibody (Cat. P42567-1) was purchased from Abmart (Shanghai, China). Rabbit anti-NF-κB p65 polyclonal antibody (Cat. 80979-1-RR) was purchased from Proteintech (Wuhan, China). HRP-conjugated goat anti-rabbit IgG (Cat. GB23303) and CY3-conjugated goat anti-rabbit IgG (Cat. GB21303) were obtained from Servicebio (Wuhan, China). Alexa Fluor 488-conjugated goat anti-rabbit IgG (Cat. ab150077) was obtained from Abcam (Cambridge, UK).

### Cell culture and grouping

All 16HBE cells were cultured in minimum essential medium (MEM, Cat. PM150410, Procell, Wuhan, China) supplemented with 5.6 mM glucose and 10% fetal bovine serum (Cat. 164,210, Procell, Wuhan, China) in a humidified incubator at 37 ℃ and 5% CO_2_ for 12 h, and experiments were subsequently conducted upon complete cellular adhesion.

**Fig. 1:**
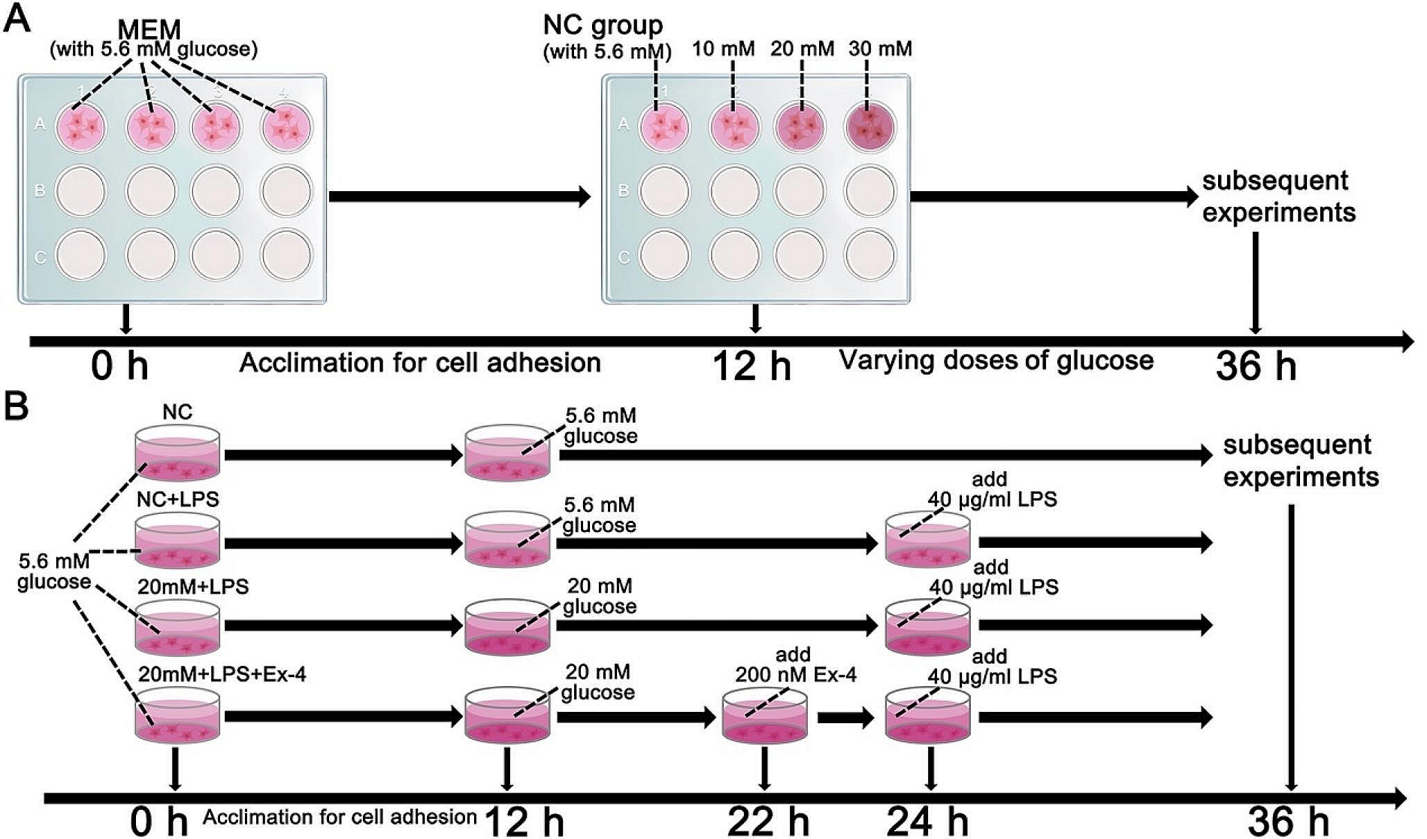
Flow charts showing the design of the present cellular experiments. (**A**) Effect of different concentrations of glucose on airway epithelial 16HBE cells. (**B**) The effects of high glucose, lipopolysaccharide (LPS), and exendin-4 (Ex-4) on 16HBE cells. MEM: minimum essential medium; NC: normal control; mM: mmol/l

First, for determination of the appropriate concentration of glucose for T1R2/T1R3, the cells were randomly grouped as follows (Fig. 1A): (1) the normal control (NC) group (with 5.6 mM glucose), (2) the 10 mM glucose group, (3) the 20 mM glucose group, and (4) the 30 mM glucose group. The glucose concentrations reported for all groups were the final concentrations including the known amount in the media used. All cells were cultured in an incubator at 37 ℃ for 24 h before the subsequent experiments.

Afterward, to investigate the effects and mechanisms of high glucose on LPS-induced airway epithelial inflammation, the cells were randomized into the following groups (Fig. 1B): (1) the normal control (NC) group (with 5.6 mM glucose for 24 h); (2) the NC + LPS group (after 12 h of preintervention with 5.6 mM glucose, 40 μg/ml LPS was added for an additional 12 h); (3) the 20 mM + LPS group (after 12 h of preintervention with 20 mM glucose, 40 μg/ml LPS was added for an additional 12 h); and (4) the 20 mM + LPS + Ex-4 group (after 10 h of preintervention with 20 mM glucose, 200 nM exendin-4, as previously described [29], was added for 2 h prior to 12 h of LPS exposure).

### Cell viability assay

The ideal concentration of LPS isolated from PA was determined by a CCK-8 assay according to the manufacturer’s protocols. Briefly, 200 μl of 16HBE cell suspension at a density of 1 × 10^4^ cells/ml was seeded into each well of a 96-well plate (Servicebio, Wuhan, China). Then, the cells were exposed to LPS at various concentrations (2.5, 5, 10, 20, 40, 80, 160, and 320 μg/ml) and maintained in a culture environment at 37 ℃ with 5% CO_2_. Each group had 5 replicate wells. After the intervention for 12 h, 10 μl of CCK-8 reagent was added to each well, and the cells were incubated at 37 °C for 1 h. Finally, the optical density (OD) of each well was measured at a wavelength of 450 nm using a microplate reader (Synergy HTX, BioTek, USA). Cell viability was assessed by the OD value.

### Cellular immunofluorescence

Each group of cells was washed three times with phosphate-buffered saline (PBS) and then fixed at room temperature with 4% paraformaldehyde for 15 min. The cells were not permeabilized to reduce accidental staining of the cell nucleus. Afterward, the cells were blocked at room temperature with goat serum for 1 h. After the goat serum was removed, the cells were incubated with primary antibodies against T1R2 (dilution, 1:200), T1R3 (dilution, 1:200) or NOD1 (dilution, 1:200) at room temperature for 1 h. After being washed three times with PBS for 5 min each time, the cells were incubated with an Alexa Fluor 488-conjugated secondary antibody (dilution, 1:400) at room temperature for 1 h and with DAPI (Cat. C1006, Beyotime Biotechnology, Shanghai, China) for 5 min. After stained cells were imaged via confocal laser scanning microscopy (FV3000, Olympus, Japan), three images were selected for each group with different fields of view. The mean gray value was then analyzed using ImageJ software (version 1.8.0, National Institutes of Health, USA).

### Cellular NF-κB activation-nuclear translocation assay

An NF-κB activation-nuclear translocation assay kit was used to assess NF-κB activation by detecting the translocation of the major subunit p65 into the nucleus through immunofluorescence staining. Notably, all reagents and antibodies used later came with the kit and did not require additional dilution. According to the assay kit protocol, after one wash with PBS, the cells were fixed with the fixative for 15 min and subsequently blocked with blocking solution for 1 h. Then, the cells were incubated with an NF-κB p65 primary antibody overnight at 4 ℃. After three 5‑min washes with the washing solution, the cells were incubated with secondary antibodies labeled with Cy3 at room temperature for 1 h. Finally, DAPI was used to stain the nuclei at room temperature for 5 min. After the stained cells were imaged using confocal laser scanning microscopy (FV3000, Olympus, Japan), three images were selected for each group with different fields of view. The mean gray value was then analyzed using ImageJ software (version 1.8.0, National Institutes of Health, USA).

### Bacterial strains and culture conditions

The *Pseudomonas aeruginosa* (PA) strain ATCC27853 was obtained as a generous gift from the Department of Laboratory Medicine of the First Affiliated Hospital of Hainan Medical University. As previously described [30], the bacteria were cultivated in Luria broth at 37 ℃ for 6 h until reaching the midlogarithmic phase. The bacteria were then extracted by centrifugation at 1500 × g for 15 min, washed twice in 0.9% NaCl without pyrogen, and resuspended in 10 ml of 0.9% NaCl. A total of 10^6^ colony forming units (CFU) per ml of PA suspension was used for intratracheal instillation.

### Animal model preparation and grouping

**Fig. 2:**
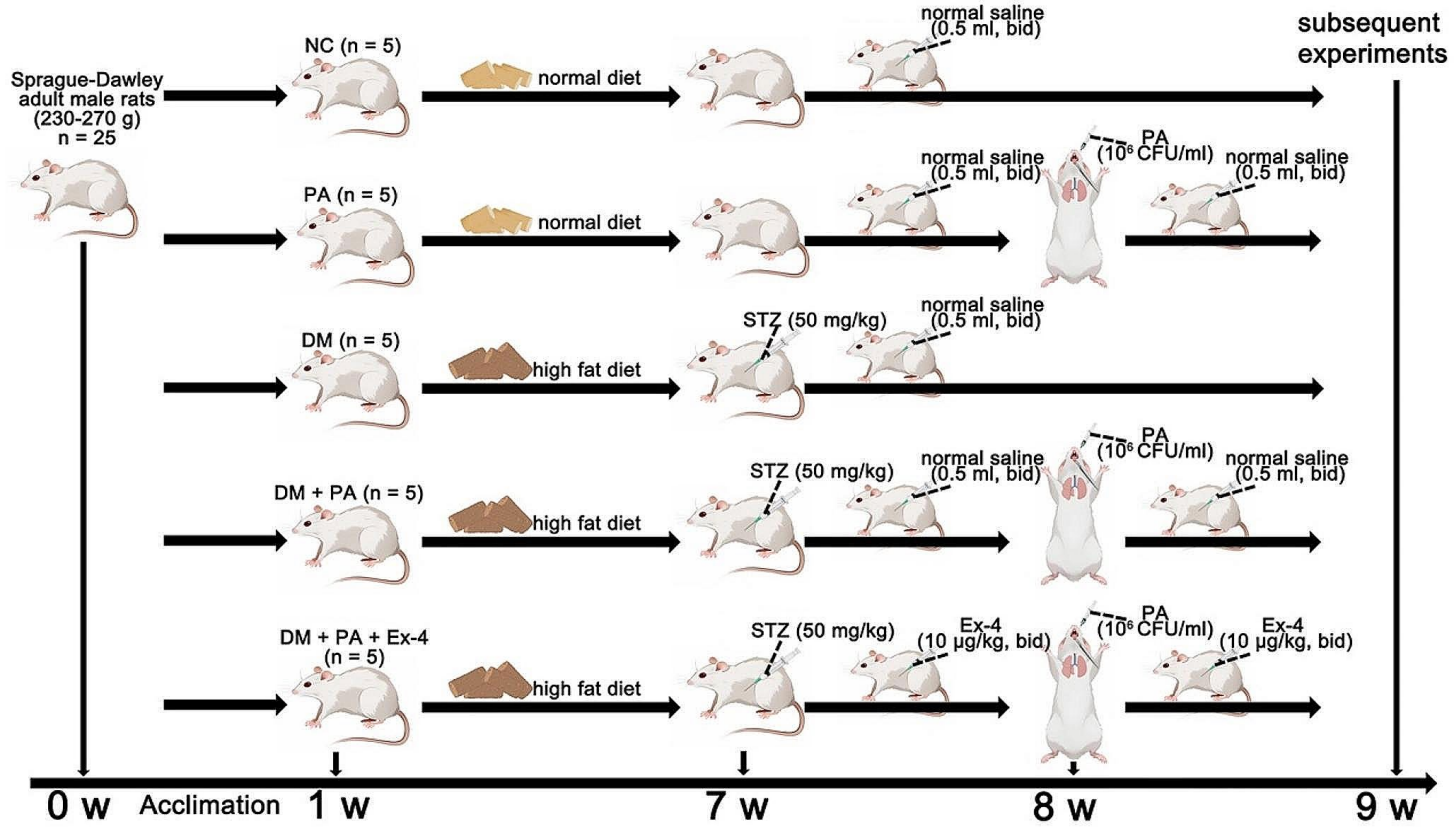
The diabetes mellitus (DM) model, *Pseudomonas aeruginosa* (PA) pulmonary infection model and PA-infected DM model were established, as was the exendin-4 (Ex-4) treatment process. NC: normal control; STZ: streptozotocin; CFU: colony forming units

Sprague‒Dawley (SD) adult male rats (230–270 g) were purchased from Hunan Slack Jingda Company (Hunan, China). The rats were provided unlimited access to food and fresh water. The rats were acclimated to a room temperature of 22–25 ℃, and three rats were housed per cage. A light/dark cycle of approximately 12 h was maintained. All in vivo procedures adhered to the National Institutes of Health (NIH) guidelines and received approval from the Ethical Committee of Hainan Medical University (Code: HYLL-2021-218). The 25 SD rats were randomly divided into five groups as follows: (1) the normal control (NC) group (with a normal diet); (2) the PA group, in which the rats were anesthetized with an intraperitoneal injection of ketamine (20 mg/kg) and subsequently given an intratracheal instillation of 50 μl of bacterial suspension, as previously described [31]; (3) the diabetes mellitus (DM) group, in which the rats were fed a high-fat diet composed of 45% fat, 35% carbohydrates and 20% protein for 6 weeks, followed by an intraperitoneal injection of streptozotocin (50 mg/kg), with high blood glucose levels exceeding 250 mg/dl as the criteria for diabetes diagnosis, as previously described [32]; (4) the DM + PA group, in which rat models were initially established for diabetes and subsequently for PA-related pneumonia; and (5) the DM + PA + Ex-4 group, in which after being induced with diabetes, rats were initially pretreated with 10 μg/kg EX-4 intraperitoneally twice a day for 7 days, as previously described [33], then infected by PA, and finally treated with the same dose of exendin-4 for another 7 days (Fig. 2).

### Bronchoalveolar lavage fluid (BALF) and lung tissue preparation

As previously described [34], all rats were anesthetized through the intraperitoneal administration of ketamine (20 mg/kg), after which a thoracotomy was performed. After the left lung bronchioles were ligated with surgical sutures, 3 ml aliquots of normal saline were slowly infused into the lungs through tracheostomy and then withdrawn gently. This process of lavage was reiterated thrice utilizing the same syringe. The pooled BALF was centrifuged at 500×g at 4 °C for 5 min. The cell-free supernatants were stored in microcentrifuge tubes at -80 °C for subsequent ELISA analysis. Finally, the left lungs were inflated and fixed with 10% formalin overnight at room temperature for subsequent experiments, and the right lungs were preserved in liquid nitrogen.

### Hematoxylin and eosin (HE) staining

The left lung tissues of the rats were collected and fixed with 10% formalin. Subsequently, the tissues were dehydrated, embedded in paraffin, and sliced into thin 5 μm slices. These sections were then stained with hematoxylin and eosin. Finally, the pathological alterations in the tissues were evaluated using a section scanner (Pannoramic MIDI, 3DHISTECH, Hungary).

### Immunofluorescence staining of lung tissue paraffin sections

As previously described [35], the lung tissue paraffin sections were deparaffinized and rehydrated, followed by antigen repair. Then, the slides were blocked with 5% donkey serum at room temperature for 30 min. Primary antibodies against T1R2 (dilution, 1:100), T1R3 (dilution, 1:100), NOD1 (dilution, 1:100) or NF-κB p65 (dilution, 1:250) were incubated with the slides at 4 ℃ overnight. Then, the slides were placed in PBS and shaken 3 times for 5 min each time. After that, the sections were incubated with secondary antibodies conjugated to Alexa Fluor 488 (dilution, 1:400) or CY3 (dilution, 1:300) at room temperature for 50 min in the dark. DAPI solution was added dropwise, and the cells were incubated at room temperature for 10 min in the dark. Finally, the slides were observed using a fluorescence microscope (Eclipse C1, Nikon, Japan).

### ELISAs of cellular and BALF supernatants

The levels of TNF-α, IL-1β and IL-6 in the extracted cellular and BALF supernatants were evaluated using the corresponding kits according to the instructions. The OD was assessed using a microplate reader (Synergy HTX, BioTek, USA) at a wavelength of 450 nm.

### Reverse transcription‑quantitative PCR (RT‑qPCR) of cells and lung tissues

Total RNA was extracted from cells and lung tissues using the TaKaRa MiniBEST Universal RNA Extraction Kit following the manufacturer’s protocols. After that, reverse transcription was performed using the TaKaRa PrimeScript RT Reagent with gDNA Eraser kit. Finally, the mRNA expression levels of TNF-α, IL-1β and IL-6 were measured using a TaKaRa TB Green Premix Ex Taq II kit. The primer sequences, which were designed using Primer Premier 5.0 software, are displayed in Table 1. The mRNA levels were quantified using the 2^‑ΔΔCq^ method [36]. All groups had at least 3 repetitions.

**Table 1:** Sequences of primers used in the present study

Gene	Primer	Sequence (5’-3’)	Accession number
Homo β-actin	Forward	CCCTGGAGAAGAGCTACGAG	NM_001101.5
	Reverse	CGTACAGGTCTTTGCGGATG	
Homo TNF-α	Forward	CCCATGTTGTAGCAAACCCTC	NM_000594.4
	Reverse	AGAGGACCTGGGAGTAGATGA	
Homo IL-1β	Forward	CGACCACCACTACAGCAAGG	NM_000576.3
	Reverse	ATGGACCAGACATCACCAAGC	
Homo IL-6	Forward	TTCGGTCCAGTTGCCTTCTCCC	NM_000600.5
	Reverse	CCAGTGCCTCTTTGCTGCTTTC	
Rat β-actin	Forward	CTTCCAGCCTTCCTTCCTGG	NM_031144.3
	Reverse	AGAGCCACCAATCCACACAG	
Rat TNF-α	Forward	TCAGTTCCATGGCCCAGAC	NM_012675.3
	Reverse	GTTGTCTTTGAGATCCATGCCATT	
Rat IL-1β	Forward	CCCTGAACTCAACTGTGAAATAGCA	NM_031512.2
	Reverse	CCCAAGTCAAGGGCTTGGAA	
Rat IL-6	Forward	GGATACCACCCACAACAGA	NM_012589.2
	Reverse	GAAACGGAACTCCAGAAGAC	

TNF‑α, tumor necrosis factor-α; IL‑1β, interleukin 1β; IL‑6, interleukin 6

### Western blot analysis of cells and lung tissues

Total protein was extracted from the cells and lung tissues using RIPA buffer supplemented with PMSF. The quantification of total protein was conducted using a BCA assay, followed by the separation of proteins on a 5–10% SDS‒PAGE gel. A volume of 20 μL of protein was sampled from each sample. Equal quantities of protein from each sample were subsequently transferred onto PVDF membranes. The membranes were then blocked with PBS with 0.05% Tween-20 and 5% nonfat milk for 1 h. Thereafter, the membranes were incubated with primary antibodies against T1R2 (diluted 1:2000), T1R3 (diluted 1:2000), NOD1 (diluted 1:2000), NF-κB p65 (diluted 1:5000) or β-actin (diluted 1:5000) at 4 ℃ overnight. After three washing cycles, the membranes were incubated with an HRP-conjugated secondary antibody (diluted 1:5000) for 2 h at room temperature. The protein bands were visualized after an additional three washes using an enhanced chemiluminescence reagent. The protein expression levels were quantified using ImageJ software (version 1.8.0, National Institutes of Health, USA), with β-actin serving as the loading control.

### Statistical analysis

All the data are presented as the mean ± standard deviation, and at least 3 repetitions were performed. The various groups were statistically analyzed through one-way analysis of variance (ANOVA), which was subsequently followed by Tukey’s post hoc test. All the data were analyzed using GraphPad Prism 9 (GraphPad Software, USA). *p* < 0.05 was considered to indicate a statistically significant difference.

## Results

### Cell viability

**Fig. 3:**
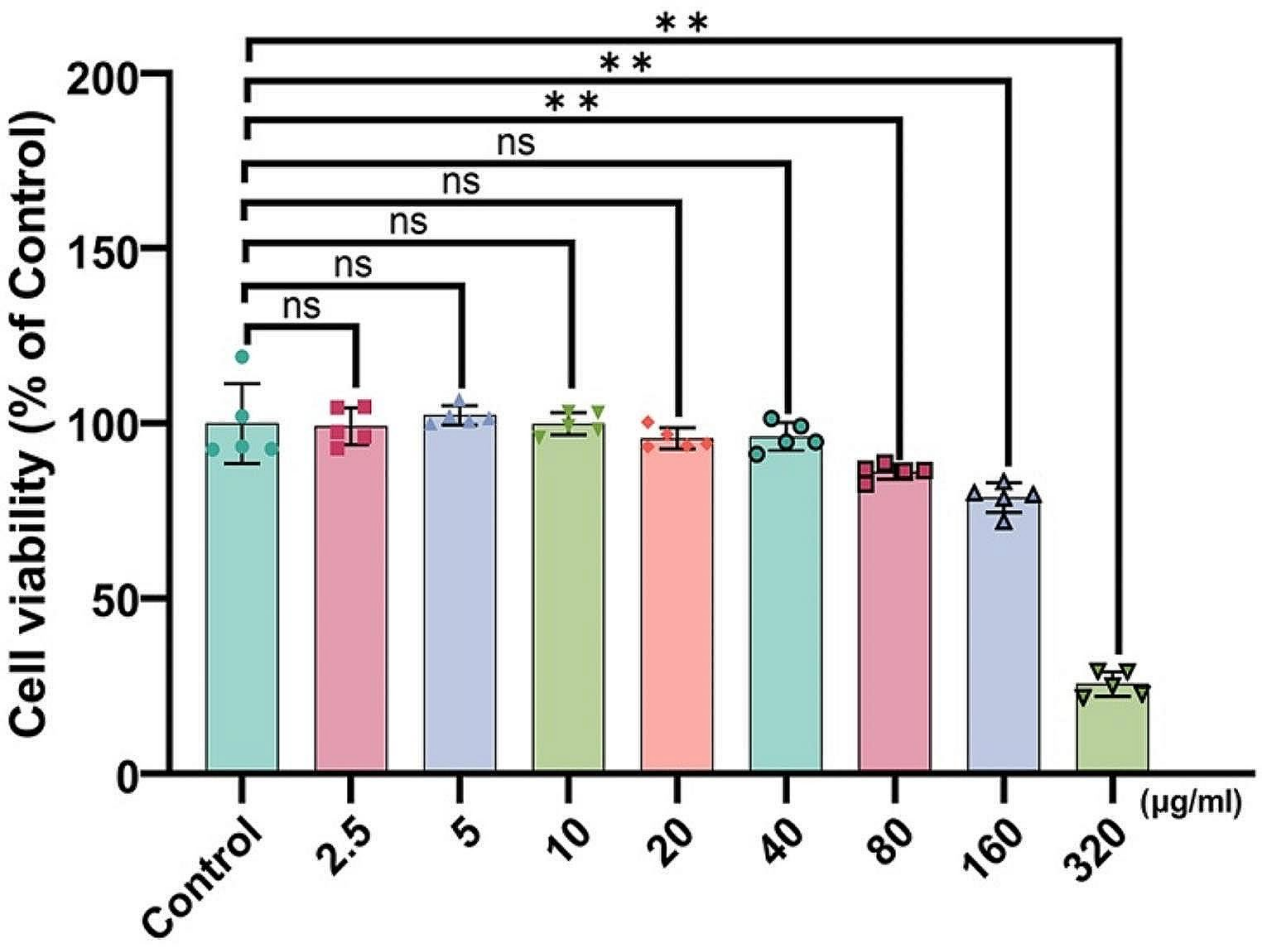
Cell viability was assessed using a cell counting kit-8 assays. Cells were exposed to various concentrations of LPS (control without LPS, 2.5, 5, 10, 20, 40, 80, 160, or 320 μg/ml) for 12 h. Data are presented as the mean ± SD (*n* = 5). ns: not significant; ^**^p < 0.01

To determine suitable concentrations of LPS isolated from PA, we treated 16HBE cells with LPS at various concentrations (2.5, 5, 10, 20, 40, 80, 160, and 320 μg/ml) for 12 h. Cell viability was subsequently evaluated using a CCK-8 assay. The assay results indicated that the groups exposed to a range of LPS concentrations from 2.5 to 40 μg/ml showed no notable difference in the cell viability levels compared to those of the control group. In contrast, a notable reduction in cell viability was observed in the groups exposed to LPS concentrations ranging from 80 to 320 μg/ml compared to the control group. The findings of the present study suggested no apparent toxicity to 16HBE cells when exposed to LPS concentrations up to 40 μg/ml (Fig. 3). Therefore, we chose 40 μg/ml as the LPS concentration for this study.

### The effects of different concentrations of glucose on cellular T1R2/T1R3

**Fig. 4:**
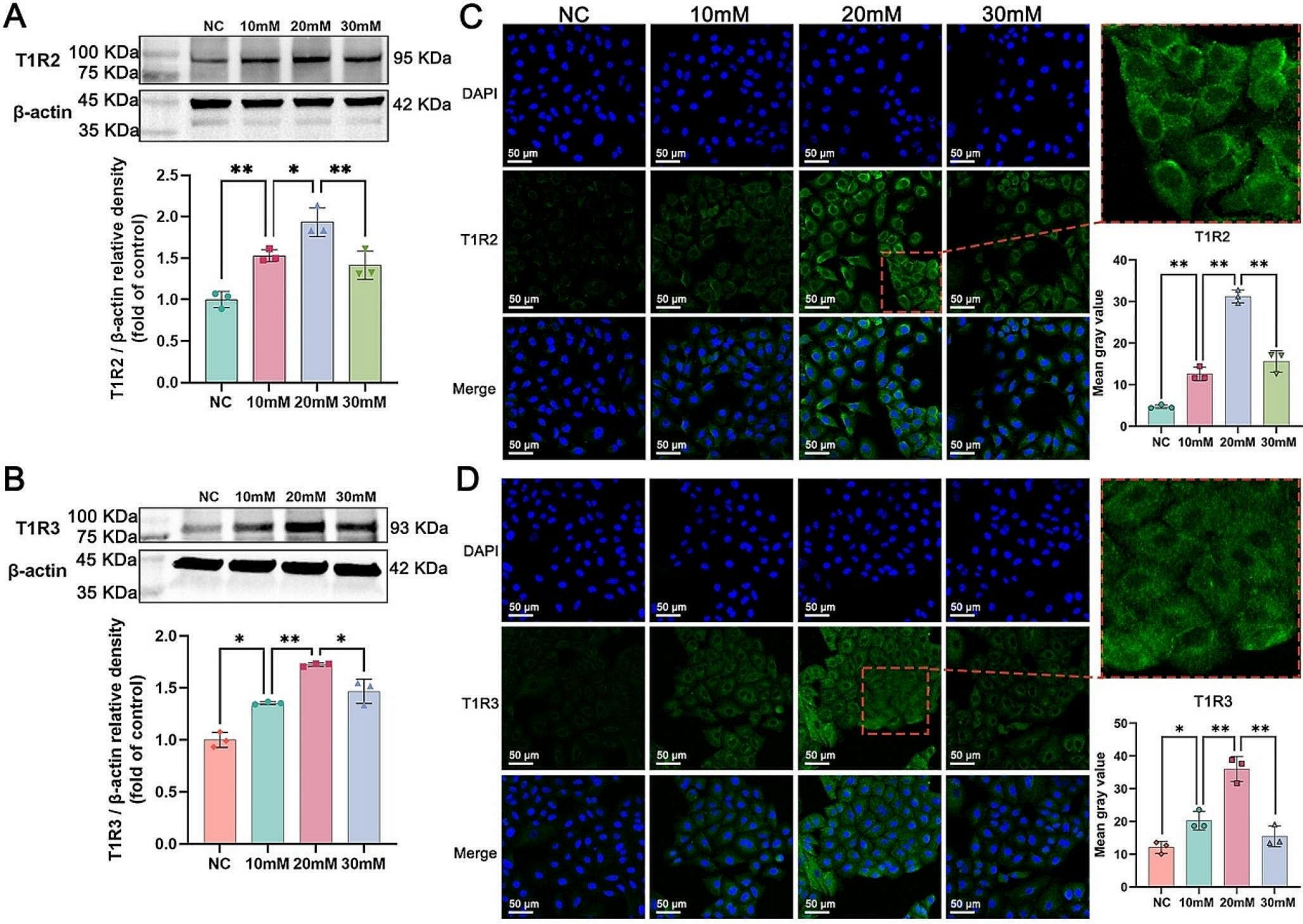
The effects of different concentrations of glucose (5.6 mM, 10, 20 and 30 mM) on the expression of T1R2 and T1R3 in 16HBE cells. Western blot quantification of (**A**) T1R2 and (**B**) T1R3 proteins relative to β-actin. Cellular immunofluorescence images of (**C**) T1R2 and (**D**) T1R3 cells were acquired using confocal laser scanning microscopy at 40× magnification (scale bar = 50 μm), and the mean gray values were computed using ImageJ software. The data are presented as the means ± SDs (*n* = 3). ^*^p < 0.05 and ^**^p < 0.01. NC: normal control; mM: mmol/l

To determine the appropriate glucose concentration for cellular T1R2/T1R3, we incubated 16HBE cells with different concentrations (5.6, 10, 20 and 30 mM) of glucose and divided the cells into 4 groups, namely, the normal control (NC), 10 mM, 20 mM and 30 mM groups. As shown in Fig. 4A and B, the western blot results revealed that the protein expression of T1R2 and T1R3 in the 10 mM group was greater than that in the NC group. The 20 mM group exhibited increased expression of these proteins compared to the 10 mM group, but decreased expression was observed in the 30 mM group compared to the 20 mM group, suggesting that the stimulatory effect of 20 mM glucose on T1R2 and T1R3 was strongest. In addition, the immunofluorescence findings indicated that T1R2 and T1R3 were primarily located in the cell membrane and cytoplasm of 16HBE cells, and similar to the western blot results, the fluorescence intensity of T1R2 and T1R3 in the 20 mM group was the most pronounced (Fig. 4C and D). Thus, we selected 20 mM as the glucose stimulation concentration for this study.

### High glucose can exacerbate LPS-induced airway epithelial inflammation, and this effect may be reversed by exendin-4

**Fig. 5:**
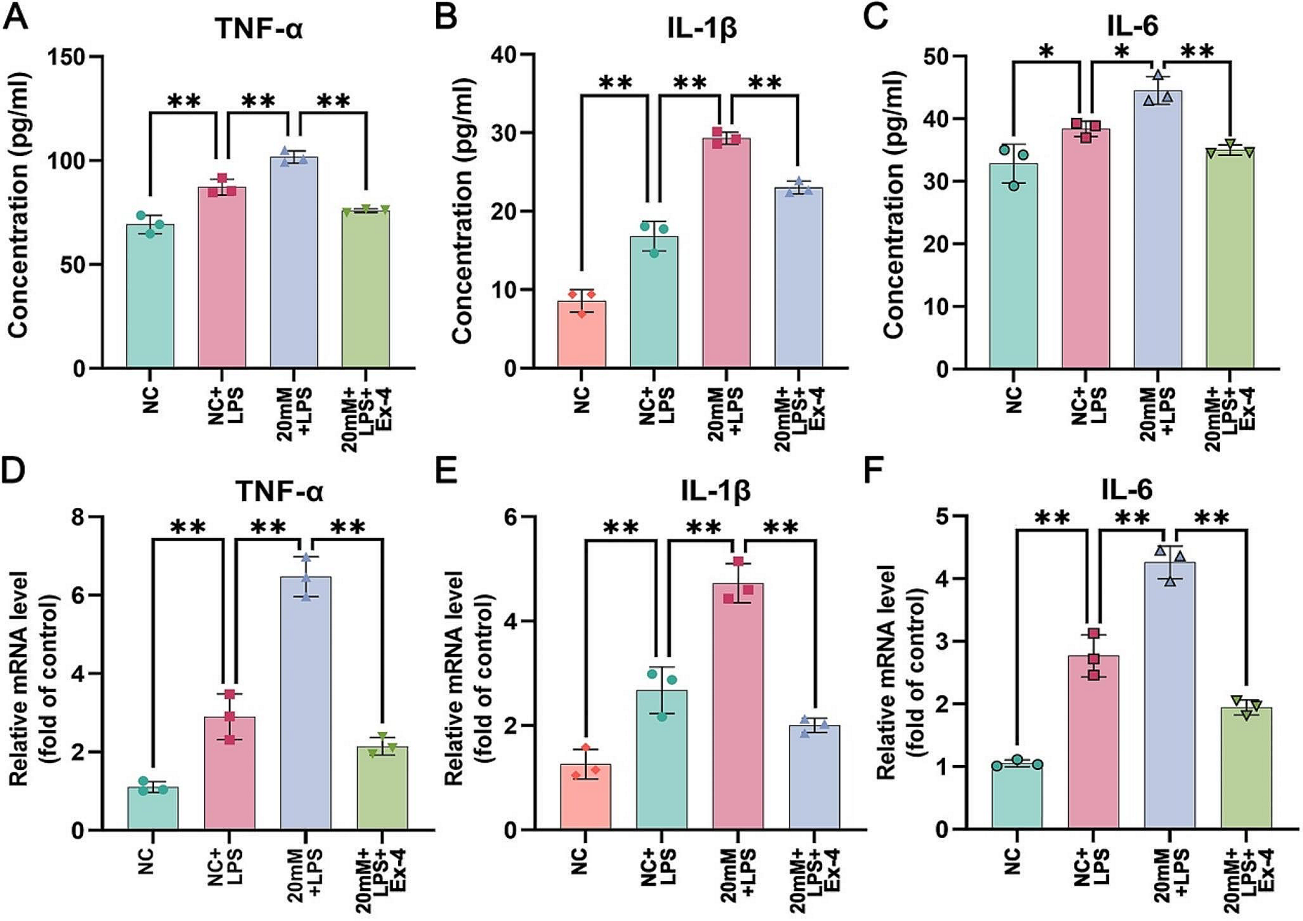
The secretion levels of (**A**) TNF-α, (**B**) IL-1β and (**C**) IL-6 in 16HBE cell supernatants were determined by ELISAs. The mRNA expression levels of (**D**) TNF-α, (**E**) IL-1β and (**F**) IL-6 relative to that of β-actin in 16HBE cells were measured by RT‒qPCR. The data are presented as the means ± SDs (*n* = 3). ^*^p < 0.05 and ^**^p < 0.01. NC: normal control; LPS: lipopolysaccharide; Ex-4: exendin-4; mM: mmol/l

As shown in Fig. 5A-C, compared with the NC (with 5.6 mM glucose), stimulation with 40 μg/ml LPS for 12 h significantly elevated the secretion of proinflammatory cytokines (TNF-α, IL-1β and IL-6), as shown by ELISAs. Notably, in an environment containing 20 mM glucose, the secretion of proinflammatory cytokines induced by LPS substantially increased compared to that in a 5.6 mM glucose environment, suggesting that high glucose could worsen airway inflammation. Conversely, upon pretreatment with 200 nM exendin-4, the LPS-induced secretion of proinflammatory cytokines in a 20 mM glucose environment was significantly reduced. Moreover, the relative mRNA expression levels of proinflammatory cytokines across different groups, as determined by RT‒qPCR, were found to be consistent with the ELISA results. As shown in Figs. 5D-F and 20 mM glucose increased the LPS-induced increase in the mRNA levels of TNF-α, IL-1β and IL-6, and this effect was reversed by exendin-4.

### Exendin-4 attenuated the overactivation of the NOD1/NF-κB pathway and the upregulation of T1R2/T1R3 stimulated by high glucose and LPS

**Fig. 6:**
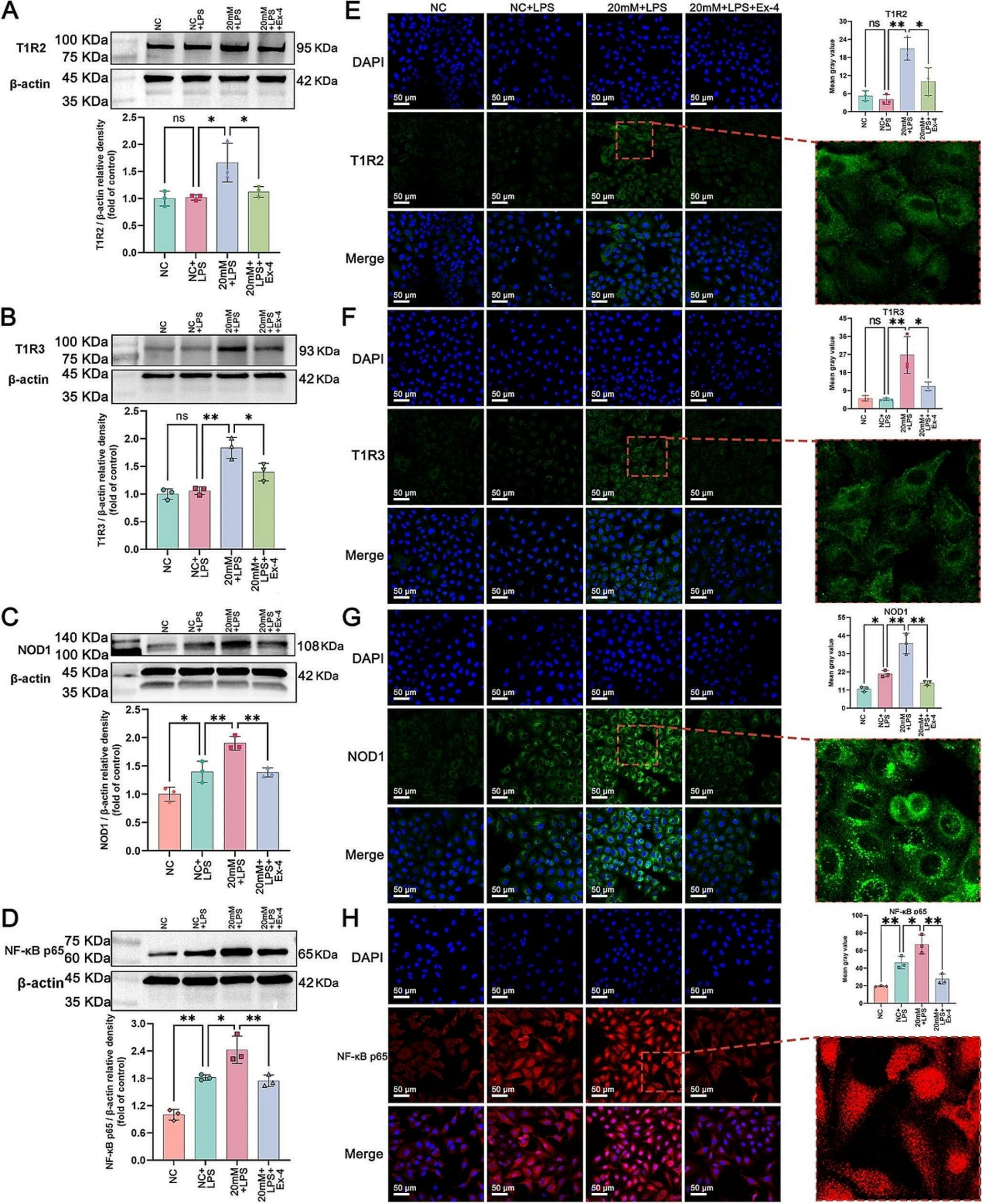
Western blot quantification of (**A**) T1R2, (**B**) T1R3, (**C**) NOD1 and (**D**) NF-κB p65 protein expression relative to that of β-actin. Cellular immunofluorescence images of (**E**) T1R2, (**F**) T1R3, (**G**) NOD1 and (**H**) NF-κB p65 were obtained using confocal laser scanning microscopy at 40× magnification (scale bar = 50 μm), and the mean gray values were computed using ImageJ software. The data are presented as the means ± SDs (*n* = 3). ns, not significant; *p < 0.05 and **p < 0.01. NC: normal control; LPS: lipopolysaccharide; Ex-4: exendin-4; mM: mmol/l

To further explore the underlying mechanisms, we used western blotting and immunofluorescence to assess the protein expression of T1R2, T1R3, NOD1 and NF-κB p65 in each group. As shown in Fig. 6A-D, compared with those in the NC group, the protein levels of T1R2 and T1R3 showed no significant changes in the NC + LPS group, indicating that LPS did not substantially affect the STRs. However, the protein levels of NOD1 and NF-κB p65 in the NC + LPS group were significantly greater than those in the NC group. In the 20 mM + LPS group, a significant increase in the protein levels of T1R2 and T1R3 and a further increase in the protein levels of NOD1 and NF-κB p65 were observed compared to those in the NC + LPS group. These results suggested that high glucose may promote the STRs and elevate the LPS-induced increase in the expression of NOD1/NF-κB pathway components. Conversely, upon pretreatment with exendin-4, the protein levels of T1R2, T1R3, NOD1 and NF-κB p65 were notably decreased compared to those in the 20 mM + LPS group. Similar to the western blot results, the fluorescence intensities of T1R2 and T1R3 were comparable between the NC and NC + LPS groups, but these values were significantly greater in the 20 mM + LPS group than in the NC + LPS group; in contrast, these values were markedly lower in the 20 mM + LPS + Ex-4 group than in the 20 mM + LPS group (Fig. 6E and F). As shown in Fig. 6G, NOD1 was predominantly expressed in the cytoplasm, and its fluorescence intensity exhibited incremental increases across the NC, NC + LPS and 20 mM + LPS groups. However, a significant decrease in the NOD1 fluorescence intensity was observed in the 20 mM + LPS + Ex-4 group compared to the 20 mM + LPS group. In essence, NF-κB functions as a transcription factor that, upon activation, migrates to the nucleus to control the expression of proinflammatory cytokines [37]. Hence, assessing p65 subunit expression in the nucleus using a specialized kit can indicate the activation-nuclear translocation of NF-κB. As shown in Fig. 6H, in the NC, NC + LPS and 20 mM + LPS groups, a persistent increase in the nuclear expression of NF-κB p65, along with a consistent increase in fluorescence intensity, was observed. However, these effects were significantly diminished in the 20 mM + LPS + Ex-4 group compared to the 20 mM + LPS group.

### Effect of exendin-4 on morphological changes in diabetic rats with pneumonia

**Fig. 7:**
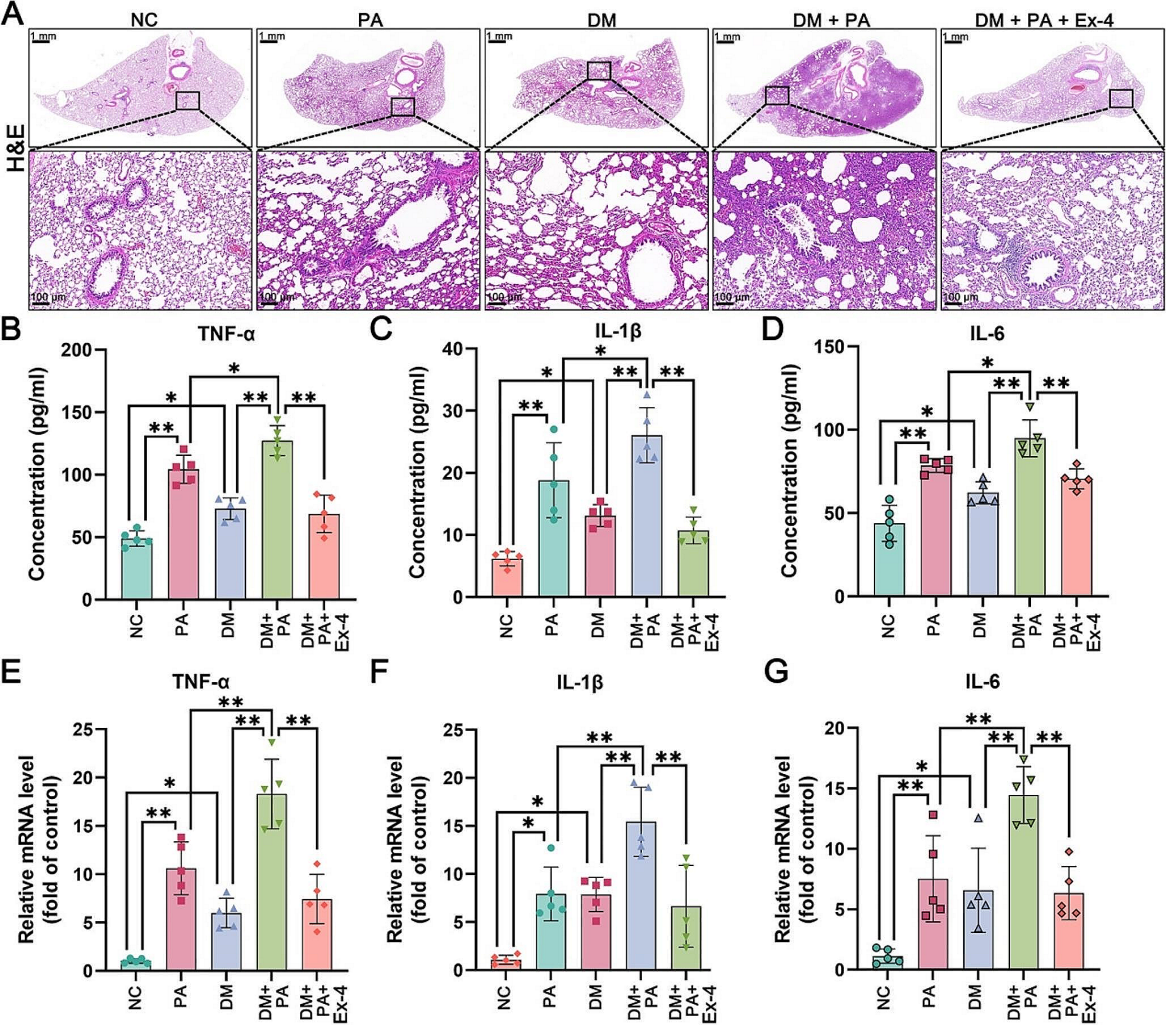
(**A**) The results of HE staining of rat lung tissues; scale bar = 100 μm. The secretion levels of (**B**) TNF-α, (**C**) IL-1β and (**D**) IL-6 in bronchoalveolar lavage fluid supernatants were detected by ELISAs. The mRNA expression levels of (**E**) TNF-α, (**F**) IL-1β and (**G**) IL-6 relative to that of β-actin in rat lung tissues were measured by RT‒qPCR. The data are presented as the means ± SDs (*n* = 5). *p < 0.05 and **p < 0.01. HE: hematoxylin and eosin; NC: normal control; PA: *Pseudomonas aeruginosa*; DM: diabetes mellitus; Ex-4: exendin-4

The main characteristics of the inflammatory response are the infiltration of inflammatory cells and pulmonary edema [38]. As shown in Fig. 7A, compared with those in the NC group, both PA infection and diabetes promoted the infiltration of inflammatory cells into the alveoli and bronchus, as well as pulmonary edema. Notably, inflammatory cell infiltration and pulmonary edema were more severe in the DM + PA group than in the PA or DM group. Conversely, treatment with exendin-4 significantly reduced these morphological changes. The results of HE staining suggested that diabetes may exacerbate the inflammatory response to lung infections, which could be mitigated by exendin-4.

### Exendin-4 reduced the overproduction of proinflammatory cytokines in diabetic rats with pneumonia

ELISAs of the proinflammatory cytokines in BALF showed that in the PA and DM groups, the levels of TNF-α, IL-1β and IL-6 were significantly greater than those in the NC group (Fig. 7B-D). In the PA + DM group, these proinflammatory cytokine levels were markedly greater than those in the PA or DM group, suggesting that diabetes may lead to the overproduction of proinflammatory cytokines during PA infection. However, compared with those in the PA + DM group, the levels of TNF-α, IL-1β and IL-6 in the exendin-4-treated group decreased significantly. In addition, RT‒qPCR was used to determine the mRNA levels of proinflammatory cytokines in the lung tissues of each group. As shown in Fig. 7E-G, the mRNA levels of TNF-α, IL-1β and IL-6 in the PA and DM groups were obviously greater than those in the NC group but lower than those in the DM + PA group. Exendin-4 treatment significantly inhibited the mRNA expression of these proinflammatory cytokines.

### Exendin-4 repressed the overexpression of NOD1/NF-κB p65 and the upregulation of T1R2/T1R3 in diabetic rats with pneumonia

**Fig. 8:**
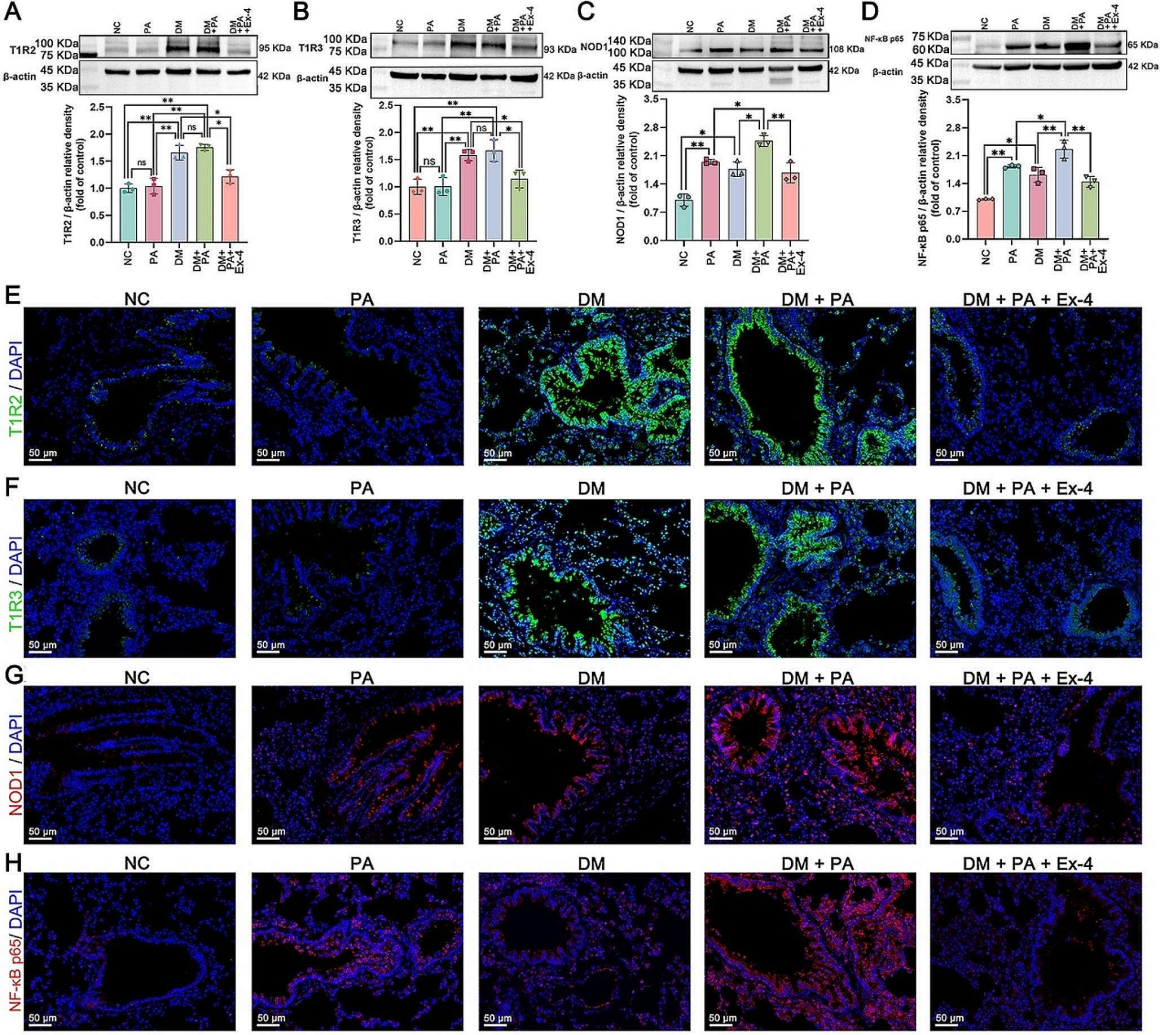
Western blot quantification of (**A**) T1R2, (**B**) T1R3, (**C**) NOD1 and (**D**) NF-κB p65 protein expression relative to that of β-actin in rat lung tissues. Immunofluorescence staining images of (**E**) T1R2 (green), (**F**) T1R3 (green), (**G**) NOD1 (red) and (**H**) NF-κB p65 (red) in paraffin-embedded rat lung tissue sections; scale bar = 50 μm. The data are presented as the means ± SDs (*n* = 3). ns, not significant; *p < 0.05 and **p < 0.01. NC: normal control; PA: *Pseudomonas aeruginosa*; DM: diabetes mellitus; Ex-4: exendin-4

To investigate the effect of exendin-4 on the expression of T1R2, T1R3, NOD1 and NF-κB p65 in the lung tissues of diabetic rats with pneumonia, we performed western blotting and paraffin immunofluorescence staining. As shown in Fig. 8A and B, no evident changes in T1R2 or T1R3 protein levels were observed between the NC and PA groups or between the DM and DM + PA groups, indicating that PA may not interfere with STR expression. However, in both the DM and DM + PA groups, the protein levels of T1R2 and T1R3 were obviously increased compared to those in the PA and NC groups. In contrast, compared with the DM and DM + PA groups, the exendin-4 treatment group exhibited decreased protein levels of T1R2 and T1R3. Furthermore, the protein levels of NOD1 and NF-κB p65 in both the PA and DM groups were greater than those in the NC group (Fig. 8C and D). Nevertheless, compared with those in the DM + PA group, significant decreases in these protein levels were observed in the DM + PA + Ex-4 group (Fig. 8C and D). These results suggested that the hyperglycemic condition of diabetes could promote T1R2/T1R3 in the lungs, and after infection with PA, hyperglycemia may enhance the elevation of the NOD1/NF-κB pathway. Exendin-4 attenuated the overactivation of the NOD1/NF-κB pathway and repressed T1R2/T1R3. This effect may be attributable either to the hypoglycemic influence of Exendin-4 or to its direct action. Similarly, the results of immunofluorescence staining of lung tissue paraffin sections demonstrated that PA infection did not affect the intensities of T1R2 and T1R3, but DM increased these intensities. (Fig. 8E and F). Both PA and DM increased the fluorescence intensities of NOD1 and NF-κB p65, and PA combined with DM further increased these intensities (Fig. 8G and H). Conversely, exendin-4 dramatically decreased the intensities of T1R2, T1R3, NOD1 and NF-κB p65 (Fig. 8E-H).

## Discussion

The present study represents the initial exploration of how exendin-4 alleviates LPS-induced inflammation in human airway epithelial cells and PA-related pneumonia in diabetic rats. The results of this study demonstrated that hyperglycemic conditions increase the production of proinflammatory cytokines (TNF-α, IL-1β and IL-6) in LPS-stimulated human airway epithelial cells and the lungs of rats with PA-related pneumonia and diabetes, along with increased activation of the NOD1/NF-κB pathway and an increase in the T1R2/T1R3. Moreover, exendin-4 improved the overexpression of NOD1/NF-κB signaling, alleviating the excessive production of proinflammatory cytokines. Exendin-4 may inhibit the T1R2/T1R3 sweet taste receptor by relieving hyperglycemia.

Diabetes has long been acknowledged as the prevailing endocrine disorder and an important contributor to demographic mortality in industrialized nations [39]. This condition can have many comorbidities, including retinopathy, nephropathy and cardiovascular diseases. However, pulmonary abnormalities were often disregarded in the past as subclinical symptoms in diabetic individuals [40]. Fortunately, the lung is now receiving increased attention as a target organ of diabetes [41]. Several studies have revealed that diabetes can reduce various lung functions, such as lung volume, the ventilatory response to hypoxia and hypercapnia, and respiratory muscle strength [42–45]. Nevertheless, the mechanisms that contribute to lung injury in diabetic patients are incompletely understood. An accepted perspective is that nonenzymatic glycosylation of proteins, induced by hyperglycemia, renders collagen more resistant to degradation and consequently causes its accumulation in pulmonary connective tissues [46]. Other researchers have hypothesized that inadequately controlled glucose may trigger the dysregulation of inflammatory responses, potentially leading to impaired lung function. A cross-sectional study revealed that diabetic patients with poor glucose management may have increased levels of inflammatory markers, including TNF-α and C-reactive protein [47]. Similarly, the present study showed that, compared with those in the lungs of normal rats, the levels of proinflammatory cytokines, inflammatory cell infiltration and pulmonary edema in the lungs of diabetic rats were greater. Additionally, diabetic patients are susceptible to pulmonary infections and prone to severe pneumonia episodes, which has always been a troubling issue for clinicians. In particular, the exacerbation of coronavirus disease 2019 (COVID-19) caused by poorly controlled diabetes has led researchers to focus on the interaction between diabetes and lung health [48]. A recent study suggested that in SARS-CoV-2-infected diabetic mice, diabetes may suppress the immune response and exacerbate inflammation, resulting in more severe lung damage [49]. This result is highly consistent with the results of the present study, which revealed that in the lungs of rats with PA-related pneumonia with diabetes, the production of proinflammatory cytokines, inflammatory cell infiltration and pulmonary edema were more severe. Recently, several in vitro investigations have demonstrated that high glucose can potentiate inflammation induced by LPS in diverse cells, including macrophages [50] and mesangial cells [51]. Similar results were observed in the airway epithelial cells of the present study. In summary, uncontrolled inflammatory responses provoked by hyperglycemia are important complications of diabetes. However, the underlying signaling molecules involved have not yet been elucidated.

Taste signaling serves as a pivotal determinant of ingestive behaviors and is posited to have a significant correlation with the regulation of metabolic processes [52]. Findings from both intra- and extraoral taste signaling pathways suggest that taste receptor systems could be therapeutic targets [53]. The obligate heterodimer formed by T1R2 and T1R3, a G protein-coupled receptor that mediates sweet taste in the oral cavity, has been found to be involved in glucose perception, glucose transporter expression and maintenance of glucose homeostasis [54]. Numerous studies have demonstrated that in diabetic organs, such as the stomach, pancreas and gut, T1R2/T1R3 expression can be significantly elevated [55]. The present study also revealed that T1R2/T1R3 expression increased in human airway epithelial cells following high glucose stimulation and in the lungs of diabetic rats. These results suggest that T1R2/T1R3 may play an important role in diabetes. In addition, several studies have investigated the role of T1R2/T1R3 in infections of the upper airway. Mucociliary clearance and the production of NO and other antimicrobial agents are important components of upper airway defense against pathogens. Ciliary beating can remove bacteria directly. NO diffuses quickly into bacteria, such as PA, destroying intracellular components [56]. However, these defensive effects are inhibited in a dose-dependent manner by sugars, including glucose and sucrose [18]. The reason for this phenomenon may be that the activation of T1R2/T1R3 could inhibit bitter taste receptors and dampen Ca^2^^+^ signaling [57]. A recent study showed that inhibition of T1R3 with Lactisole significantly enhanced NO production in basal airway epithelial cells [19]. These results may explain the susceptibility of the airway to pathogens in diabetes. Nevertheless, the effects of pathogens on T1R2/T1R3 have not been elucidated. Recently, two studies showed that LPS could reduce T1R2/T1R3 expression in the lingual epithelium, as well as T1R3 expression in the pulmonary endothelium [17, 58]. However, the results of the present study showed that LPS did not significantly affect the expression of T1R2/T1R3 in airway epithelial cells. This finding may be related to the dose, duration and cell type of the LPS intervention, which requires further research. Acyl homoserine lactones (AHLs) secreted by gram-negative bacteria can promote the expression of bitter taste receptors, which may repress T1R2/T1R3 [59]. However, bacterial D-amino acids can increase T1R2/T1R3 expression [60]. These results indicated that individual components of the pathogen differentially influence the expression of T1R2/T1R3, which could explain why PA had no effect on T1R2/T1R3 expression in the rat lungs in our present study. Certainly, we cannot eliminate the possibility that the observed result is associated with the concentration of PA and the duration of infection. At present, the functions of T1R2/T1R3 in the lower respiratory tract and in modulating the inflammatory response are not well characterized. The results of a recent study indicated that inflammation-related mRNA expression was significantly decreased in the colon of T1R3 knockout mice [28]. Similarly, the present study implied that elevated T1R2/T1R3 may be related to the exacerbation of LPS-induced airway epithelial inflammation in a high-glucose environment and lung inflammation in PA-infected diabetic rats. In short, the role of T1R2/T1R3 in diabetic lung infections deserves further investigation, and its downstream signaling pathways are not yet known.

The recognition of certain molecules in pathogens by pattern recognition receptors (PRRs) is an essential step in initiating proper host defense mechanisms [61]. However, PRRs are double-edged swords because their overexpression can exacerbate inflammation and damage cells. NOD1, an intracellular PRR, is induced by various stimuli, including live and heat-killed bacteria, interferon-γ and TNF-α [62]. In this study, both LPS and PA promoted the expression of this gene. Currently, NOD1 is believed to activate NF-κB to induce the expression of proinflammatory cytokines, which are crucial in bacterial infections. However, recent reports suggest that NOD1 has important functions not only in bacterial infections but also in nonbacterial infections and insulin resistance [63]. NOD1 is upregulated in the myocardium of diabetic humans and mice [64]. The results of one study showed that high glucose can enhance the expression of the NOD1/NF-κB pathway in LPS-stimulated mesangial cells [51]. These results indicate the importance of NOD1 in diabetes, but its role in the diabetic lung is still unclear. Our present study suggested that in the lungs of diabetic rats, NOD1/NF-κB was significantly upregulated. Moreover, the upregulation of NOD1/NF-κB was further increased in airway epithelial cells stimulated with high glucose and LPS, as well as in the lungs of PA-infected diabetic rats. However, the interaction between NOD1 and T1R2/T1R3 requires further research.

Clinically, reasonable control of glucose has always been the key to the treatment of diabetic patients with lung infection. Recent studies have shown that several hypoglycemic drugs also have anti-inflammatory properties [65]. Exendin-4 can reduce blood glucose by activating GLP-1R and has been found to alleviate inflammation in various diabetic organs, including the colon and testis, via the inhibition of NF-κB [66]. The results of a randomized controlled trial demonstrated that exendin-4 can reduce the levels of inflammatory markers in the blood of patients with T2DM [67]. However, the role of exendin-4 in diabetic patients with lung infection has rarely been reported, and its anti-inflammatory mechanism is unclear. The results of the present study showed that exendin-4 significantly alleviated the exacerbation of LPS-induced airway epithelial inflammation in a hyperglycemic environment and the worsening of PA-related pneumonia in diabetic rats. This therapeutic effect may be due to the attenuation of NOD1/NF-κB overexpression and the inhibition of T1R2/T1R3. Additionally, exendin-4 might suppress T1R2/T1R3 either as a consequence of hypoglycemia or through a direct mechanism that requires further research.

The present study also had several limitations. First, the data of this study are largely descriptive, associative and correlative, with no direct proof that the T1R2/T1R3 mediate inflammatory signal activation in the lung, so in the future we should employ T1R2/T1R3 knockout models in airway epithelial cells or rats to sufficiently elucidate the regulatory role of T1R2/T1R3 in airway inflammatory responses and the NOD1/NF-κB pathway. Second, the absence of certain critical groups in this study makes it unclear whether exendin-4 directly inhibits the T1R2/T1R3 and NOD1/NF-κB pathways. Third, the sample size of this study is small, indicating the need for larger sample sizes for future research to increase the robustness of the findings. Fourth, we focused exclusively on PA, a quintessential example of a gram-negative bacterium, to delineate diabetes complicated with pulmonary infection. Further research is needed to extend our understanding of other pathogenic infections with diabetes.

## Conclusions

The present study suggested that in lung infection with diabetes, high glucose can aggravate the respiratory inflammatory response, increase the elevation of NOD1/NF-κB, and promote T1R2/T1R3. Exendin-4 can ameliorate PA-related pneumonia with diabetes and overexpression of NOD1/NF-κB. Additionally, exendin-4 might suppress T1R2/T1R3, potentially through its hypoglycemic effect or through a direct mechanism. The relationship between the inhibition of T1R2/T1R3 and the remission of PA-related pneumonia with diabetes, as well as the reduction in the NOD1/NF-κB pathway, warrants further research.

## Data Availability

The data and materials employed in the present study are accessible from the corresponding authors upon reasonable request.

## Abbreviations

DM: Diabetes mellitus
Ex-4: Exendin-4
GLP-1R: Glucagon-like peptide-1 receptor
IL-1β: Interleukin-1β
IL-6: Interleukin-6
LPS: Lipopolysaccharide
NF-κB: Nuclear factor-κB
NOD1: Nucleotide-binding oligomerization domain-containing protein 1
PA: *Pseudomonas aeruginosa*
PRRs: Pattern recognition receptors
STR: Sweet taste receptor
T1Rs: Taste 1 receptors
TNF-α: tumor necrosis factor-α
